# Towards DNA-Based Methods Analysis for Honey: An Update

**DOI:** 10.3390/molecules28052106

**Published:** 2023-02-23

**Authors:** Sónia Soares, Francisca Rodrigues, Cristina Delerue-Matos

**Affiliations:** REQUIMTE/LAQV, ISEP, Polytechnic of Porto, Rua Dr. António Bernardino de Almeida, 4249-015 Porto, Portugal

**Keywords:** honey, DNA-based methods, DNA metabarcoding, environmental DNA

## Abstract

Honey is a natural product widely consumed all over the world due to its relationship with healthy benefits. Additionally, environmental and ethical issues have a higher weight in the consumer’s choice to buy honey as a natural product. Following the high demand of this product, several approaches have been suggested and developed aiming at the assessment of honey’s quality and authenticity. Target approaches, such as pollen analysis, phenolic compounds, sugars, volatile compounds, organic acids, proteins, amino acids, minerals, and trace elements, showed an efficacy, particularly concerning the honey origin. However, a special highlight is given to DNA markers, due to their useful applicability in environmental and biodiversity studies, besides the geographical, botanical, and entomological origins. Different DNA target genes were already explored for addressing diverse sources of honey DNA, with DNA metabarcoding attaining a relevant importance. This review aims to describe the latest advances on DNA-based methods applied in honey related studies, identifying the research needs for the development of new and additional required methodologies, and to select the most adequate tools for future research projects.

## 1. Introduction

**Figure 1:**
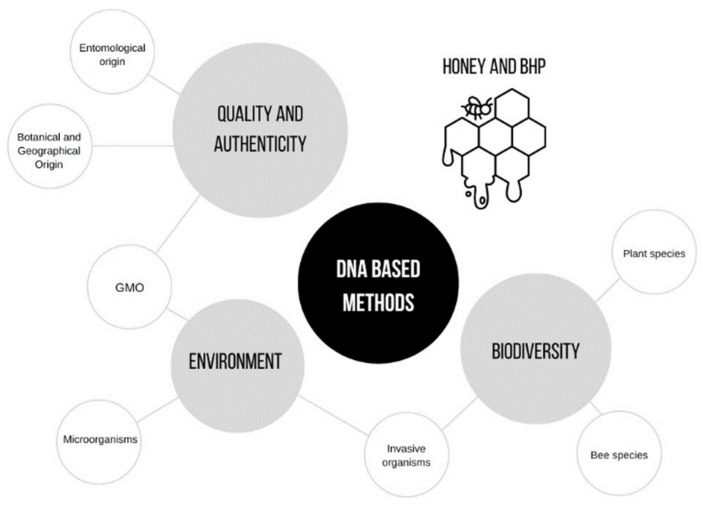
Different applications of DNA-based methods in honey and BHP, according to the literature referred in the present review.

In the previous years, the population’s concern with health and well-being has led to a rising interest in natural foods, such as plants, fruits, or their derived products. Additionally, consumers are more conscious of the environmental global problems, bearing in mind environmental and ethical issues in the moment of choice. Altogether. This contributes to a high demand for biological enriched natural foodstuffs, such as honey. It is known that honey is consumed worldwide since our ancestors, due to its taste, nutritional value, and health benefits. This natural product is produced by honey bees, which also provide pollen, propolis, royal jelly, wax, and bee bread. These beehive products (BHPs) have been highly sought after, with a high importance by the consumers and industries due to its highlighted biological properties [1]. Despite honey being mainly composed of water and carbohydrates (namely fructose and glucose), other minor compounds are also present, such as vitamins, enzymes, volatile compounds, minerals, and amino acids, which are responsible for the specific/individual organoleptic and nutritional properties, as well as its biological effects, such as antibacterial, hepatoprotective, hypoglycemic, antihypertensive, gastroprotective, antifungal, anti-inflammatory, and antioxidant, among others [2,3,4,5,6,7,8]. This composition is closely linked to the plants of the surrounding area of beehives visited by honey bees during honey production [2,9]. The biologic compounds of plants are present in the nectar collected and are transferred to honey, contributing for the individuality of each honey concerning the organoleptic, nutritional, and biological properties [2]. In that sense, the botanical and geographical origins of honey cannot be neglected. Monofloral honeys and honeys with a Protected Designation of Origin (PDO) designation are generally perceived as high-quality products, being preferred by consumers and, consequently, achieving higher economic values [2]. Therefore, they are more susceptive to adulterations and fraud. Numerous procedures, based on target (pollen analysis, phenolic compounds, sugars, volatile compounds, organic acids, proteins, amino acids, minerals and trace elements, and DNA markers) and nontarget approaches, were presented, aimed at the assessment of honey authenticity, particularly regarding the origin [2]. Concerning the use of DNA markers for honey authentication, the employment of DNA-based methods have been showing promising results and high potential for botanical and entomological origin identification of honey. In the previous years, a growing research interest in DNA-based methods in honey and BHP have been observed for environmental and biodiversity studies (Figure 1) [10,11,12,13,14,15,16,17].

The DNA present in honey is derived from different sources (plants, virus, bacteria, microorganisms, and honey bees) that honey bees are in contact with during foraging activities for nectar collection and environmental exploration. Additionally, honey bees also are in contact with the environmental contaminants present in the surrounding areas of beehives. During the return to the honeycomb to deposit nectar, honey bees transfer traces of these organisms and contaminants to the honeycomb and honey, which represent an increased risk for the survival of beehives [18]. Some authors classified honey bees as environmental sentinels that accumulate contaminants (e.g., pesticides, polycyclic aromatic hydrocarbons (PAHs), heavy metals, antibiotics, plastic-related chemicals) from local sources of pollution (e.g., urban traffic emissions, forest fires, agricultural activity) in their tissues and incorporate them into BHPs [10,19]. Recent evidence suggests a huge impact of the environmental pollution on honey bees and BHPs [19,20,21,22,23,24].

## 2. Methodology

Specific information on the topic was collected from the literature available from search engines such as Google Scholar, PubMed, Science Direct, Scopus, and Web of Science for retrieving published data (from 2010 to 2022) using different combinations of keywords, i.e., DNA-based methods, honey, honey authenticity, honey botanical origin, entomological origin of honey, and honey DNA, among others. The inclusion criteria were limited to full text articles on DNA-based approaches applied to honey and beehive products. Seventy-one papers were included in these criteria and have been revised considering the focus of this paper.

## 3. DNA-Based Methods

In recent years, the world has assisted in the substantial growth of DNA-based methods and its wide range of applications (Table 1). DNA-based methods are used in different areas, such as disease diagnosis and drug development through molecular identification, phylogenetic inference, genetic and genomic analysis, assessment of authenticity of food products regarding quality and ethical issues through species identification, variety/breed differentiation, origin authentication, and allergy detection, as well as in the study of quality environments through environmental DNA studies [17,25,26,27,28,29,30,31,32,33].

The need for fast, simple, and accurate techniques led to the persistent research and development of improved DNA-based techniques. Therefore, these methods have become increasingly relevant for food products authentication and quality-related issues, whose challenges are diverse and may include the identification of individuals, species, breeds, cultivar, or varieties in animals, plants, fungi, and microorganisms [34]. Since DNA is present in all biological tissues, it can potentially be recovered from any matrix containing even a very small number of cells or cell debris [34]. In eukaryotic organisms, DNA is stored in the cell nucleus and in the organelles, such as mitochondria and chloroplasts [34]. Moreover, DNA has a high chemical and thermal stability, making this molecule resistant to food processing and severe environmental conditions [2]. Usually, mitochondrial DNA (mtDNA) is well suited for origin studies in animals, while plastid DNA is suggested for plants, although nuclear DNA can also be used. The high number of copies of mtDNA and plastid DNA per cell is a significant advantage when analysing sub-optimal samples, such as processed foodstuffs [34].

For complex matrices such as honey, DNA-based methods were an important development, especially regarding its authenticity and quality assessment, based mostly on honey’s botanical origin determination [2,11,12,25,31,35]. Melissopalynology is the conventional approach to identify the botanical origin of honey and obtain information about its geographical origin. However, this is a laborious and time-consuming technique that needs to be performed by analysts with considerable expertise due to the high variability of pollen morphology of some plant species [36,37]. In this sense, the development and improvement of DNA-based methods for honey and its related products were performed during the last years by different authors [11,31,36,38,39]. Recently, Chiara et al. [36] analysed several honey samples of different botanical and geographical origin through the DNA metabarcoding technique, aiming to identify honey botanical origin and verify the authenticity of the label information on the product. The DNA was tested for the amplification of the fragment *trnL* of the chloroplastidial gene through next-generation sequencing (NGS). The achieved results demonstrated the detection of plant sources at the species/genus/family level [36]. Nevertheless, the combination of multiple markers to achieve a better discrimination of taxonomical groups is suggested [40].

Another approach commonly used in honey products is the employment of DNA-based methods for entomological studies. According to the standards of the Codex Alimentarius [41], honey is the natural sweet substance produced by different species of honey bees. Among the eight different species of the genus *Apis*, *A. mellifera* and *A. cerana* are of economic importance due to their use in apiculture [32]. However, other species belonging to genus, such as Melipona, are also producers of valued honey products. The distribution of honey bees varies according to the geographical location. Therefore, the entomological origin of honey could be used as an important assessment for honey quality and authenticity, together with the botanical and geographical origin. In this sense, studies suggesting the use of DNA-based methods are emerging. Metabarcoding was used by Prosser and Hebert [39] to identify the botanical and entomological origins of honey produced by *A. mellifera* and *M. beecheii*. In addition, Kek and co-workers [42] employed DNA sequencing and forensically informative nucleotide sequencing (FINS) of the ribosomal RNA (16S rRNA) gene region and mitochondrial cytochrome c oxidase subunit I (COI) gene region to classify and identify the bee species in honey.

**Table 1:** Research developed in the last years applying DNA-based methods to honey DNA assessment studies. The table represents the DNA extraction methods selected, as well as the identification methods and target(s) selected for the respective final aim. Additionally, some values of DNA yields and purities are transcribed from the respective papers.

Application	DNA Extraction	DNA Identification	DNA Yields	A260/A280 Ratio Range	Target	Reference
**Botanical Origin**	DNeasy^®^ Blood and Tissue Kit (Qiagen)	Qualitative PCR and Real-time PCR			*adh1*, *actin*, LFY1, hmg, nr1, PAL, DXR, *Profilin*, ypr10, *trnL*	[43]
	NucleoSpin^®^ Plant (Macherey-Nagel), DNeasy^®^ Plant Mini Kit (Qiagen), CTAB-based and Wizard^®^ DNA-based	Qualitative PCR	nd–592.6 ng/uL	1.0–2.1	18S rRNA and *adh1*	[44]
	DNeasy^®^ Isolation and Purification Kit (Qiagen)	Qualitative PCR	10.0–25.0 ng/μL	∼1.80	*rbcL* and *trnH-psbA* plastid region	[45]
	DNeasy^®^ Plant Mini Kit (Qiagen)	DNA Metabarcoding			*rbcL*	[46]
	DNeasy^®^ Plant Mini Kit (Qiagen)	DNA Metabarcoding			*rbcL*	[47]
	CTAB-based Method	Next-generation Sequencing (NGS)			rbcL, *matK* and ITS2	[37]
	NucleoSpin^®^ Plant II (Macherey-Nagel)	Qualitative PCR, Real-time PCR with HRM Analysis	4.4–275.9 ng/μL	1.9–2.3	18S rRNA and *matK*	[31]
	CTAB-based Method	Ion Torrent Sequencing (NGS)			*trnL*-UAA	[11]
	DNeasy^®^ Blood and Tissue kit (Qiagen), QIAcube Instrument (Qiagen)	DNA Metabarcoding	0.1–29.7 ng/μL	0.5–2.2	*trnL*	[36]
**Pollen DNA**	Automated CTAB Buffer-based Method, Maxwell^®^ 16 FFS Nucleic Acid Extraction System, Custom-Kit (Promega GmbH), QIAQuick PCR Purification Kit (Qiagen)	Qualitative PCR, Real-time PCR	4.1–10.7 ng/μL	2.0	*actin*	[48]
	DNeasy^®^ Power Plant Pro Kit (Qiagen)	Qualitative PCR and Sequencing			ITS2	[49]
**Botanical Origin and Entomological**	In-house Method (Silica Membrane Spin Column)	Qualitative PCR and Ion Torrent Sequencing			ITS2, *rbcLa*, and COI	[39]
	CTAB-based Method	Qualitative PCR, Real-time PCR and NGS Sequencing			*trnL*-UAA, Cox1, and COI	[13]
	DNeasy^®^ *mericon*^TM^ Food Kit (Qiagen)	Qualitative PCR and Sanger Sequencing			16S rRNA and COI	[42]
**Entomological Origin**	CTAB-based Method, Wizard^®^ DNA-based and the Commercial Kits DNeasy^®^ *mericon*^TM^ Food Kit (Qiagen) and NucleoSpin^®^ Isolation Food Kit (Macherey-Nagel)	Qualitative PCR	0.1–1210.6 ng/μL	0.6–2.6	16S rRNA	[25]
	CTAB-based Method	Qualitative PCR and Sanger Sequencing			COI-COII intergenic spacer	[12]
	NucleoSpin^®^ Plant II Kit (Macherey-Nagel)	Qualitative PCR, Real-time PCR with HRM Analysis, Sanger Sequencing	2.3–303.9 ng/µL	1.1–2.6	18S rRNA, tRNA^leu^ -cox2 intergenic region, and 16S rRNA	[50]
	NucleoSpin^®^ Plant II Kit (Macherey-Nagel)	Real-time PCR with HRM Analysis and Sanger Sequencing			COI	[35]
**Geographical Origin**	Wizard^®^ DNA-based	Machine Learning (Sequencing)				[51]
**Arthropods, Plants, Fungi, Bacteria, and Viruses**	CTAB-based Method	Qualitative PCR and Ion Torrent Sequencing				[15]
**Plants, Bacteria, and Fungi**	DNeasy^®^ Plant Mini Kit (Qiagen)	DNA Metabarcoding			ITS2, *rbcLa*, *trnL*, 16S rRNA, and ITS	[38]
	Dialysis and Phenol⁄chloroform⁄isoamyl Alcohol-based Method	Qualitative PCR and Sequencing	0.07 ng ⁄μL	1.35	16S rDNA and 18S rDNA	[52]
**Pathogens and Parasites**	CTAB-based Method	Qualitative PCR and Sequencing		>1.6	COI-COII, 16S rRNA, NapA, SSU rRNA, *cytb*, 18S rRNA, and Cox1	[10]
**Viruses, Bacteria, Plants, Fungi, Protozoans, Arthropods, and Mammals**	CTAB-based Method	Shotgun Sequencing				[16]
**Parasite *Lotmaria passim***	CTAB-based Method	Qualitative PCR			*cytb*, 18S fragment and GAPDH fragment	[53]
**Genetically Modified Organism**	CTAB-based Method	DNA Concentration, DNA Integrity, PCR Amplification	0.1–0.2 ng/μL		CaMV 35S promoter, 35S and *Bt* junction gene and *Sad1* gene	[54]

The use of PCR and real-time PCR with HRM analysis for *A. cerana* and *A. mellifera* DNA identification, as well as for the differentiation of *A. mellifera* mtDNA lineages in honey samples, were performed by Soares et al. [32] and Honrado et al. [35], respectively. *A. mellifera* is considered the most important species for honey production, encompassing more than 30 subspecies and ecotypes [12]. In Europe, the native subspecies belong to three different lineages: A (e.g., *A. m. iberiensis* and *A. m. siciliana*), M (e.g., *A. m. iberiensis* and *A. m. mellifera*), and C (e.g., *A. m. ligustica* and *A. m. carnica*) [50]. Utzeri and their coworkers [12] developed a method based on the mtDNA lineage which was able to identify the *A. mellifera* subspecies. With the specific primers developed, they were able to distinguish honey produced by *A. m. siciliana* (A lineage) from *A. mellifera* of the C and M lineages. Additionally, Soares et al. [55] proposed a DNA-based method aiming to authenticate the entomological origin of European honeys. The authors were able to identify *A. m. iberiensis* honeybees belonging to the A lineage (Portugal and Spain) by targeting the mitochondrial gene *cytb* and to differentiate honeybees from *A. mellifera* of C and M lineages by targeting the COI gene.

Since honey is an interesting source of environmental DNA (eDNA) signatures, information based on the DNA can be used for honey authentication, determining its entomological, botanical, and geographical origin, while also allowing one to study the quality of the honeycomb surrounding the environment and to detect/monitor the presence of invasive organisms [10,15]. Wirta et al. [38] used DNA metabarcoding and the metagenomics of plants, bacteria, and fungi extracted from the honey samples to differentiate its geographical origin. The DNA analysis of honey samples is also gaining importance for determining the origin and detecting genetically modified organisms (GMOs), microorganisms, or potentially allergenic species [36,38,48,52,54]. Olivieri et al. [52] demonstrated the feasibility of DNA analysis to detect a wide range of plants, fungi, and bacteria in honey. The authors attested that the DNA extracts provide enough DNA suitable for PCR amplification, allowing for the detection of DNA from the symbiotic bacteria of the intestinal tract of honey bees and the identification of a wide range of plant species [52]. Despite the progress and developments in these advanced and precise methodologies, the available studies on honey and it related products to detect GMOs, invasive microorganisms (such as fungi, bacteria, and virus), or potentially allergenic species are still scarce.

### 3.1. DNA Extraction Methods

Despite the promising use of DNA-based methods in a wide range of applications, several factors present in processed or natural food products must be taken into consideration which may affect the success and accuracy of these methods [44,56]. The food processing or the absence of treatments in natural foods can contribute to the degradation of DNA into smaller fragments or into a large amount of non-specific DNA in the product, which complicates the DNA analysis. In addition, the presence of food matrix components that are considered as interferents in DNA analysis can inhibit the PCR amplification [2,41,56]. Nevertheless, the analysis of the genetic material of plant/animal material from honey and related products requires previous work and the selection of an adequate DNA extraction method. DNA extraction is an essential routine step in DNA-based protocols, being necessary as an efficient extraction with high-quality DNA, yield, and good purity [44]. A low integrity and purity of DNA may reduce the successful PCR amplification of the targeted DNA regions, particularly the amplification of relatively long regions [56]. The high-quality DNA is characterized by high molecular weight fragments, with an A260/280 ratio between 1.8 and 2.0 [57]. Some difficulties appear in the DNA analysis of plants and natural products due to the presence of several cell components, including polysaccharides, proteins, and DNA polymerase inhibitors, such as alkaloids and polyphenols [57]. In addition to that, there are plants with specific characteristics in cell structures that complicate the DNA extraction. In the specific case of pollen grains (the main source of plant DNA in honey), the difficulties are related with size, structure, and quantity of pollen in honey samples [58]. The double wall of the pollen grain, allied to the waxes and proteins contained on the surface, hinders the brake of the grain wall, affecting the successful DNA extraction. Additionally, large quantities of pollen could release greater amounts of DNA polymerase inhibitors, affecting the PCR performance [58].

A previously sampled treatment for the extraction of genetic material is imperative in the case of honey and its related products. The developed studies presented methods based on centrifugation, filtration, or a combination of both [49]. Aimed at the use of DNA-based methods for botanical species identification to distinguish monofloral honeys, Soares et al. [44] tested three different sample pre-treatments, including 1: phosphate-buffered saline solution (PBS) and centrifugations; 2: ultrapure water, centrifugations, and ultrasonic baths; 3: ultrapure water, centrifugations, and glass beads. Additionally, the authors combined each pre-treatment with five DNA extraction methods (the NucleoSpin^®^ Plant II Kit (methods A and B) (Macherey-Nagel, Düren, Germany) and DNeasy^®^ Plant Mini Kit (Qiagen, Mississauga, Canada), and the in-house CTAB-PVP based and Wizard methods). The results demonstrated a wide range of DNA yield and purity, depending on the previous treatment and the DNA extraction method. According to the authors, the Wizard method attained the highest yield values, with the best results achieved in combination with the treatment that included a mechanical pollen disruption step (glass beads) (312.5–592.6 ng/µL, for pre-treatment 3) when compared to the other pre-treatments (193.2–382.7 ng/µL and 22.7–109.9 ng/µL, for pre-treatment 2 and pre-treatment 1, respectively). Nevertheless, the CTAB-PVP and DNeasy methods were also successful in the specific amplification of alcohol dehydrogenase 1 (adh1) gene of *Calluna vulgaris* in the heather honey. Similar results were obtained by Soares et al. [31,32] using severe variations of temperature during the pre-treatment of honey samples. The frost/defrost process proved to be equally efficient at attaining, after NucleoSpin^®^ Plant II DNA extraction, a range of yields of 4.4 to 275.9 ng/μL and 2.3–303.9 ng/µL [31,32]. In a study of DNA extraction methods comparisons, Kek et al. [25] performed honey samples pre-treatment based on the dilution of honey with PBS, a water bath at 40 °C, and centrifugations. The authors achieved promising results, namely a yield of 1210.6 ng/μL with NucleoSpin^®^ Plant II DNA extraction method [25]. The high yield attained is probably due to the stronger buffering capacity of PBS to separate superior amounts of sugars from honey [25].

Over the years, different DNA extraction methods have been suggested for the DNA extraction from honey [25,48,54]. Conventional DNA extraction methods are based on the use of cetyltrimethylammonium bromide (CTAB) and sodium dodecyl sulphate (SDS) [44]. However, these methods involve the use of hazardous chemicals, such as phenol or chloroform, during DNA extraction [25]. On the other hand, commercial DNA extraction kits, based on silica columns or magnetic beads that bind to DNA, are less hazardous and time-consuming and, simultaneously, more cost effective [59].

Different DNA extraction methods were employed in honey origin studies, such as the commercial DNeasy^®^ Blood and Tissue Kit (Qiagen, Hilden, Germany) [43], DNeasy^®^ Plant Mini Kit (Qiagen, Mississauga, ON, Canada) [44], DNeasy^®^ Mericon Food Kit (Qiagen, Mississauga, Canada) [25], DNeasy Isolation and Purification Kit (Qiagen, Hilden, Germany) [45], NucleoSpin Plant Kit II (Macherey-Nagel, Duren, Germany) [31,44], NucleoSpin^®^ Isolation Food Kit (Macherey-Nagel, Duren, Germany) [25], and in-house developed protocols, such as CTAB-PVP-based and Wizard^®^ DNA-based (Promega Corporation, Madison, WI, USA) [32,46,60]. Kek et al. [25] compared four DNA extraction methods, including CTAB-based method, Wizard^®^ DNA-based and the commercial DNeasy^®^ Mericon Food Kit (Qiagen, Mississauga, ON, Canada) and NucleoSpin^®^ Isolation Food Kit (Macherey-Nagel, Duren, Germany), to evaluate the honey origin. According to the authors, the DNeasy method achieved the best performance regarding the evaluated parameters (DNA concentration and purity, PCR amplification capacity targeting mitochondrial 16S rRNA gene, and execution time). Despite several developments in accomplishing the best combination of pre-treatment and DNA extraction methods, the yields obtained from the honey DNA extraction could be affected by high amounts of phenolic compounds and polysaccharides present in the plants of origin, which are transferred to honey, compromising the enzymatic activity of DNA polymerase in PCR [61].

In a study performed by Prosser and Herbert [39], the results obtained for the botanical and entomological assessment proved that the sequence recovery is more difficult for dark or flavoured honeys [39]. The authors compared the number of operational taxonomic units (out), detected for the *rbcLa* gene, from dark, medium, and light honey samples. The DNA from the liquid extract of dark honeys revealed four botanical sources, while the DNA from the light honeys revealed 55 OTUs from different DNA sources, including honey bee DNA [39].

The colour and flavour of honey are related with the presence of phenolic compounds, with darker honeys usually showing higher contents than lighter ones [62]. Thus, the Prosser and Herbert [39] results perfectly reflect the inhibition by plant secondary compounds on PCR [39].

### 3.2. DNA Identification Approaches

DNA-based methods are used in honey studies mostly to identify pollen as an alternative to melissopalynology. However, a set of PCR approaches (PCR sequencing, PCR-restriction fragment length polymorphism (PCR-RFLP), and species-specific PCR) for the detection of relevant species from different kingdoms in honey were successfully purposed by different authors [31,43,50]. The use of the PCR technique with fluorescence probes was demonstrated to be useful in the identification of known and particular plants in honey, confirming the honey’s geographical origin [43]. Soares et al. [31] developed a novel DNA barcoding approach coupled with HRM analysis for the botanical authentication of lavender honey. The method allowed for the identification of different lavender species in three clusters (lavender species commonly found in Portugal, namely *L. stoechas* subsp., *L. pedunculata*, and *L. viridis*); the species *L. multifida* and *L. pinnata*; and the French lavender species (*L. angustifolia* and *L. latifolia*) [31]. The high potentiality of the DNA barcoding technique has been suggested as an alternative approach for botanical and entomological origin assessments [32,45,55]. Nevertheless, unlike the barcode gene suggested for animal species identification (COI gene), the identification of plant species have several difficulties associated. Although diverse genes have been tested [31,37,39,40,45,46,51,63,64], most of the results only achieve family- or genus-level identification and are dependent on sequencing analysis, either using the Sanger method or next-generation sequencing (NGS) [2]. Moreover, the lack of universality in primers, associated with the incompleteness of plant reference libraries, has been appointed as a drawback [47]. Currently, DNA metabarcoding has attained high interest for the taxonomic identification of different sources of DNA in honey [2,36,39,40,51,65]. This is a high throughput sequencing based method that relies on the PCR amplification of targeted DNA regions suggested as barcodes [51]. This technique allows for the analysis of complex samples, such as honey, which contain mixtures of species, providing an extensive depth of sequencing coverage and associated ecological insights [40,65]. Several studies have addressed a variety of research topics, contributing to advances in honey related research. Despite DNA metabarcoding having essentially been proposed for botanical and entomological assessments [11,13,32,37,39,42,45,51], studies aimed at different approaches for honey have been emerging. For example, de Vere et al. [65] and Danner et al. [66] used DNA metabarcoding in honey samples to investigate the foraging behaviour of honey bees in controlled surrounding plant resources of beehives. In a similar approach, Hawkins et al. [51] investigated the plants that were frequently used by honey bees, concluding that certain species or plant groups have particular importance in the honey bee’s environment. Regarding the significance and richness of honey as a source of eDNA, Wirta et al. [38] directed the DNA metabarcoding approach to differentiate honeys from different countries through plant, bacterial, and fungal taxa identification. However, the eDNA samples could contain several distinct species belonging to different kingdoms or phyla, attaining a high degree of complexity that could be difficult to capture or characterize using standard barcoding or metabarcoding approaches [67]. Based on this, Bovo et al. [15] applied a next-generation sequencing platform (Ion Torrent) for the shotgun metagenomic analysis of honey eDNA, a direct sequencing of eDNA samples without any PCR enrichment, confirming its potential usefulness for multi-kingdom DNA signature assessment.

#### 3.2.1. Target Genes

To study the botanical origins of honey, several barcode regions, namely, the plastidial genes *matK*, *rbcL*, and *trnL*, as well as internal transcriber spacers (ITS1 and ITS2) and intergenic spacer *trnH-psbA*, have been proposed [68,69]. However, the selection of the most adequate target gene for plant species identification in honey is a continuous challenge. The potential of *trnL* gene was investigated by different authors [11,36,39,55]. The primers trnL-g/trnL-h, developed by Taberlet et al., (2010), were used in different works to identify the botanical origin of honey samples [11,39]. The achieved results proved its applicability for studying environmental and degraded DNA [11,55], while also being, however, unable to identify some plants beyond the family level [11,39]. Although the *trnL* gene presented a high sensitivity and resolution in the identification of plant groups, the low resolution of the P6 loop at the species level contributes to the difficulty in the species distinction, since many closely related species exhibit the same P6 loop sequence [55]. The *rbcL* region and the *trnH-psbA* spacer were evaluated as DNA barcoding tools for the identification of honey botanical origins [45]. Contrary to the *trnL* marker, the *trnH-psbA* spacer has a good performance at species level identification, while for the *rbcL* region the results showed a limited ability for botanical identification, allowing for the identification of plants only at the genus level [45]. The plastidial *matK* gene was targeted by Soares et al. [31] as a candidate barcode for *Lavandula* species identification in honey samples. The specific primers designed allow one to differentiate several species in three distinct clusters with the HRM analysis. Nevertheless, with the DNA barcoding methodology, it is necessary to generate a reference library and, for that, a previous knowledge of the botanical composition of honey is imperative. Knowing these limitations, Prosser and Herbert tested three gene regions (ITS2, *rbcLa*, and COI) to simultaneously assess the information on the botanical and entomological origins of honey. The *rbcLa* gene was used to detect trace and/or degraded plant DNA in honey, from pollen or not, while the ITS2 spacer was specifically chosen to identify the pollen DNA signature, covering both possible botanical sources of plant DNA (pollen and pollen-free). The COI gene was also used to assess the entomological sources of DNA in honey samples [39]. The results obtained demonstrated that the ITS2 gene can identify plants below the family level, which is consistent with the previously results reported by Richardson et al. [40], which successfully used the ITS2 region to obtain the taxonomic identification of honey samples at the genus level.

Despite the feasibility of the identification of the plant sources in honey through DNA markers, the quantification of the plant species is still a challenge. Baksay et al. [58] investigated the relationship between the amount of pollen grains in mock solutions and the abundance of sequence reads. The authors used the ITS1 internal transcriber spacer and the plastidial P6-loop of *trnL* (UAA) intron. The results showed a strong positive relationship between the number of DNA sequences and the number of pollen grains in mock solutions. However, the DNA marker, the plant species, and the number of PCR cycles were affected by the pollen counting methodology [58]. Nevertheless, additional studies, including different plant species and plant species, mixtures are required.

Regarding entomological identification, different mitochondrial DNA fragments have been suggested [12,13,32,42,55]. The mitochondrial large subunit ribosomal RNA (16S rRNA) gene region and the mitochondrial cytochrome c oxidase subunit I (COI) gene region were suggested by Kek et al. [42] to classify and identify honey produced by *Apis* honey bees and *Trigona* stingless bees. The authors accurately identified the genetic identities of honey origins from *A. dorsata*, *A. mellifera*, *A. cerana*, and *Heterotrigona itama*. The COI gene is frequently used as the marker of choice for animal species’ identification [68], being selected by several authors for honey bee species’ identification and discrimination in honey [35,55].

**Figure 2:**
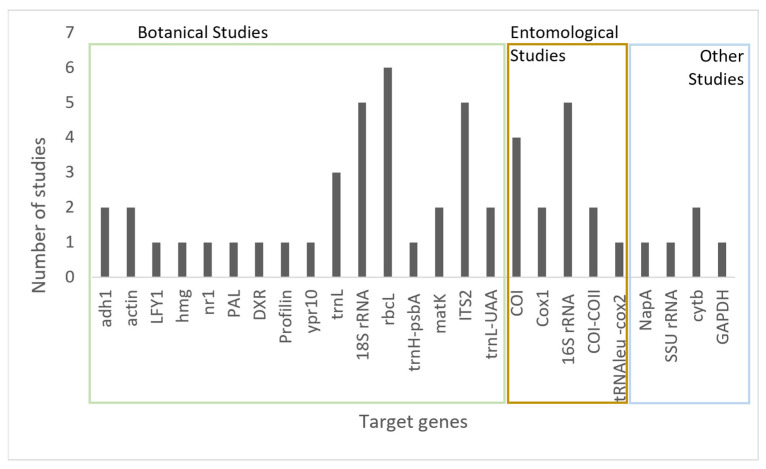
Summary of the most studied target genes in honey DNA studies and indication of the principal final application.

In a consumer health-related approach, the DNA analysis of honey samples is gaining importance for determining the origin, as well as for the detection of genetically modified organisms (GMOs) [48,54], microorganisms [52,53], or potentially allergenic species [63]. The graphic shown in Figure 2 represents a schematic summary with the most studied target genes in honey DNA studies.

#### 3.2.2. Honey Environmental DNA

Recently, special attention has been given to the foraging activity of honey bees. These pollinators have contact with different organisms during the nectar search, highlighting the importance of honey and beehive products, their relationship with the ecological system, and their role in biodiversity. Thus, honey can be a source of environmental DNA (eDNA), being possible to attain information not only about the entomological, botanical, and geographical origin, but also from possible hive co-existing organisms, such as honey bee pathogens and parasites. These activities allow one to monitor the health status of honey bee colonies and the environmental biodiversity [10,13,15,16]. Different honey eDNA-based approaches have been tested targeting different organisms, such as parasites *Lotmaria passim* [10,70], *Nosema ceranae* [10,71], and *Varroa destructor* [10,14], as well as the bacteria *Paenibacillus larvae* [10]. Recently, Bovo et al. [16] developed a novel eDNA approach consisting of the deep shotgun sequencing of eDNA coupled with a specifically adapted bioinformatic pipeline that is able to monitor several organisms from different kingdoms or phyla (viruses, bacteria, plants, fungi, protozoans, arthropods, mammals). In fact, the use of honey eDNA proved to be a suitable and simple alternative for monitoring the incidence and distribution of honey bee pathogens and parasites. However, a limited number of studies are still available and deep insights are urgently requested.

#### 3.2.3. Honey MicroRNAs

The last developments in honey genetic studies were conducted by researchers for the analysis of honey microRNAs. A study performed by Gismondi et al. [72] identified mRNA and tRNA molecules in honey samples from Europe, suggesting that honey may contain unique miRNA profiles depending on the floral source. The authors concluded that honey RNA is stable and could retain functionality, proving to be useful as molecular markers for honey botanical origins. Moreover, plant miRNAs are important molecules in the regulation of some biological functions in human beings, contributing to some of the medicinal properties of honey [72].

The analysis of these small molecules, despite recent studies, has been a promising tool for honey authentication related studies. Through NGS sequencing, Smith et al. [73] mapped an RNA profile of honey samples and identified small regulatory RNAs, including miRNAs and tRFs. Their results revealed the presence of small RNAs derived not only from plants, but also from invertebrates, bacteria, and viral species. With these findings, the authors proved that the expression of regulatory small RNA is dynamic and changes in response to specific environments and stresses [73].

## 4. Conclusions

Honey is a natural product consumed worldwide due to its taste and flavour, but mostly due to its well-documented and proven relationship with health and well-being. The consumer’s demand for this product led to quality and authenticity concerns by authorities, producers, and researchers.

Honey presents DNA derived from different sources (plant, honey bees, virus, bacteria, and other microorganisms), which makes DNA-based methods promising tools for its quality and authenticity evaluation, and for environmental and biodiversity studies. As demonstrated in this review, developments in DNA-based methods addressing different sources of honey DNA have been successfully achieved. In the complete DNA evaluation process, different factors need to be considered: a sample treatment may be necessary to obtain an efficient extraction of genetic material in order to undercut the presence of several cell components (polysaccharides, proteins, and DNA polymerase inhibitors, such as alkaloids and polyphenols) and characteristics (pollen size and structure); the species DNA (gene copy number) may interfere with an efficient extraction and, consequently, with an efficient DNA evaluation.

In general, honey DNA identification and evaluation has been reported using different PCR approaches (PCR sequencing, PCR-restriction fragment length polymorphism (PCR-RFLP), and species-specific PCR), with universal primers for barcoding, or using metabarcoding approaches. DNA metabarcoding proved to be a promising technique for honey-related studies due to its feasibility in the analysis of complex honey eDNA sources in a wide range of monitoring approaches related to honey and BHP, such as health monitoring, environmental biodiversity, and the botanical, geographical, and entomological origin of honey.

In summation, DNA-based research in honey is a continuous developing work that is important to contributing to biodiversity preservation, the sustainable conservation of honey bee species, and the assurance of consumers’ and producers’ interests.

Despite the progress obtained in the previous years, which led to the DNA metabarcoding of eDNA from honey, studies concerning specific points, such as GMO detection, invasive microorganisms (such as fungi, bacteria, and virus), or potentially allergenic species, are still highly requested.

In the last few years, the study of small RNAs, as a new approach addressing honey genetic studies, has been explored. These molecules proved to have potential as markers in honey origin assessment, since they are related with the plant status at the time that honey bees collect nectar for honey production.

## Data Availability

No new data were created or analysed in this study. Data sharing is not applicable to this article.
